# Response to trastuzumab deruxtecan and delayed immune-related events in a patient with metastatic *HER2*-positive NSCLC: a case report and literature review

**DOI:** 10.3389/fonc.2024.1469438

**Published:** 2025-01-17

**Authors:** Lu Wang, Shidi Wen, Wenjia Zhu, Zhiyang Zhang, Yuejuan Cheng

**Affiliations:** ^1^ Chinese Academy of Medical Sciences & Peking Union Medical College, Beijing, China; ^2^ Department of Nuclear Medicine, Beijing Key Laboratory of Molecular Targeted Diagnosis and Therapy in Nuclear Medicine, Peking Union Medical College Hospital, Chinese Academy of Medical Sciences & Peking Union Medical College, Beijing, China; ^3^ Department of Medical Oncology, Peking Union Medical College Hospital, Chinese Academy of Medical Sciences & Peking Union Medical College, Beijing, China

**Keywords:** antibody-drug conjugate, trastuzumab deruxtecan, immune-related adverse events, lung cancer, case report

## Abstract

Trastuzumab deruxtecan (DS-8201) is an antibody-drug conjugate (ADC) designed to target *HER2* mutations. We reported a case study demonstrating a favorable response to DS-8201 in a patient with *HER2* mutation-positive non-small cell lung cancer (NSCLC) who exhibited resistance to initial immunotherapy, along with delayed immune-related events of hypoadrenocorticism occurring five months after discontinuation of immune checkpoint inhibitors. After reviewing the relevant literature, we discussed the mechanism of ADC agents underlying their anti-tumor activity and the potential impact of DS-8201 on the tumor microenvironment. This case highlights the efficacy of DS-8201 in NSCLC, particularly in individuals who have failed first-line immunotherapy, and provide valuable insights for clinicians exploring innovative strategies for the management of patients with lung cancer.

## Introduction

*Human epidermal growth factor receptor 2* (*HER2*, also known as *ERBB2*) alterations, encompassing gene amplification, mutation, and protein overexpression, are identified in 2-3% of nonsquamous non-small cell lung cancer (NSCLC) (1). Unlike mature targeted therapies for oncogene-driven NSCLC such as *epidermal growth factor receptor* (*EGFR*) and *anaplastic lymphoma kinase* (*ALK*), there is currently no approved standard targeted therapy for NSCLC with *HER2* alterations (2). The predominant treatment is platinum-based chemotherapy, either alone or in combination with immunotherapy or antiangiogenic therapy, referring to the treatment of stage IV patients without driver mutations, with an objective response rate (ORR) remaining below 30% (3).

Inspired by research in other solid tumors and gene mutation contexts, other anti-tumor agents have been explored in the landscape of *HER2*-positive NSCLC. The combination of chemotherapy with trastuzumab, a *HER2*-targeting monoclonal antibody, did not yield significant benefits in phase II trials (4, 5). The combination of trastuzumab and pertuzumab also demonstrated an ORR of less than 30% in a limited sample size (6). The non-selective tyrosine kinase inhibitor (TKI) afatinib failed to achieve satisfactory efficacy in 13 pretreated *HER2*-positive patients in the Phase II NICHE trial (7); while poziotinib exhibited ORRs of 27.8% and 39% in two cohorts of the ZENITH20 trial, with median progression-free survival (PFS) of 5.5 months and 5.6 months (8, 9), respectively. Furthermore, pyrotinib demonstrated a 30% ORR in second-line monotherapy (10), and its combination with apatinib exhibited promising therapeutic efficacy in the PATHER2 trial (ORR 51.5%, median PFS 6.9 months) (11). Trastuzumab emtansine (T-DM1), a classic representative of antibody-drug conjugates (ADCs), demonstrated limited efficacy when guided by HER2 protein expression levels, with treatment responses observed in less than 20% of patients with immunohistochemically confirmed HER2-positive status (12). In conclusion, the current treatment requirements of the *HER2*-positive NSCLC population remain unmet, with several agents not exhibiting as satisfactory efficacy as in gastric and breast cancers or in oncogene-negative NSCLC. This underscores the urgent need for alternative therapeutic strategies (3).

The emergence of trastuzumab deruxtecan (T-DXd, also known as DS-8201) represents a potential paradigm shift. DS-8201 is a *HER2*-targeted ADC that has shown promising results in breast and gastric cancer (13, 14). Recent studies suggest that DS-8201 may also hold therapeutic potential in lung cancer patients. In particular, the open-label, multicenter phase II DESTINY-Lung 01 trial (NCT03505710) (15) in 2022 evaluated the efficacy and safety of 6.4 mg/kg DS-8201 in 91 patients with metastatic *HER2*-mutant NSCLC refractory to standard treatment. The study demonstrated an impressive ORR of 54.9%, with a median progression-free survival (PFS) of 8.2 months and a median overall survival (OS) of 17.8 months. The most common adverse event observed was interstitial lung disease (ILD), which occurred in 26% of patients. DESTINY-Lung 01 led to FDA approval of DS-8201 for *HER2*-positive NSCLC. Subsequently, the DESTINY-Lung 02 trial (NCT04644237) (16), a blinded phase II study, compared the efficacy and safety of DS-8201 at doses of 5.4 mg/kg and 6.4 mg/kg in 152 platinum-treated patients with *HER2*-mutant NSCLC, more than 70% of whom had received prior anti-PD-(L)1 therapy. ORRs and median PFS in the two dose arms were 50.0%, 10.0 months vs. 56.0%, 12.9 months, respectively, with a differential incidence of grade ≥ 3 adverse events favoring the 5.4mg/kg dose. Recently, the phase II DESTINY-Lung 05 study (NCT05246514) further confirmed the efficacy and safety of DS-8201 in the Chinese population. These findings may pave the way for improved therapeutic options in this challenging cancer subtype. However, given that DS-8201 has not yet been included in standard treatment regimens, there are currently few real-world observational studies on its efficacy and safety in lung cancer patients.

Here we report a case focusing on the use of DS-8201 in a patient with metastatic NSCLC who has demonstrated resistance to first-line immunotherapy. By delving into the mechanism of action of ADC drugs and reviewing previous research on DS-8201, this report sheds light on the potential efficacy and safety of DS-8201 as a viable treatment option for these patients.

## Case report

**Figure 1:**
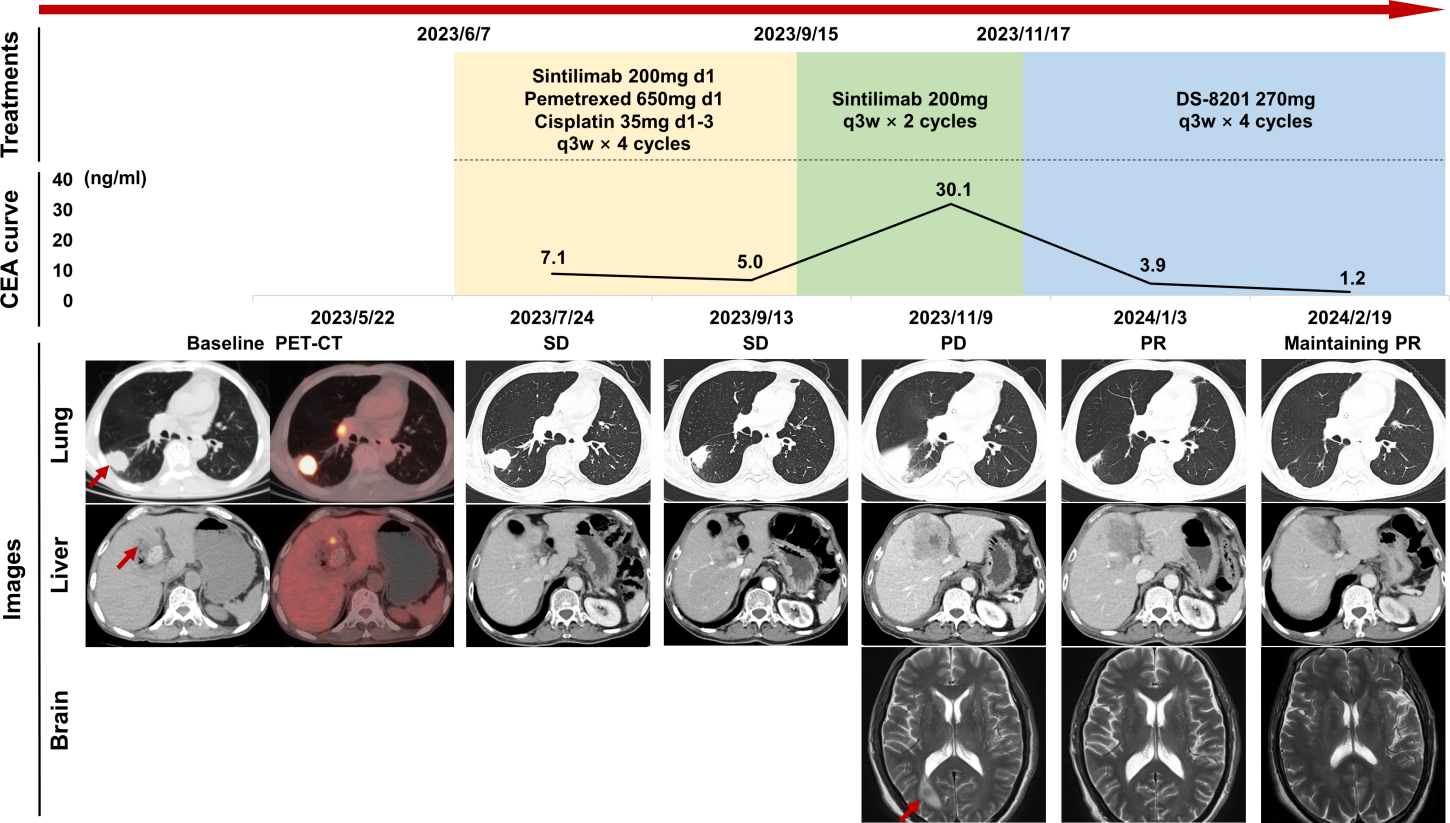
Timeline of patient treatment and efficacy assessment. The “Treatments” section shows the time course of the treatment regimens, including immunochemistry, immune maintenance, and attempt of DS-8201. The “CEA curve” section illustrates the dynamic changes in tumor marker levels over time. The “Images” section displays chest and abdominal CT scans and brain MRI images at different stages of disease development, with efficacy assessments based on RECIST criteria. Red arrows indicate tumor lesions. CEA, carcinoembryonic antigen; CT, computed tomography; MRI, magnetic resonance imaging; PD, progression disease; PET, positron emission tomography; PR, partial response; q3w, quaque 3 weeks; RECIST, response evaluation criteria in solid tumors; SD, stable disease.

The adult patient, over 60 years of age, who had smoked 40 cigarettes per day for over 20 years and had quit smoking 20 years ago, presented with symptoms of cough and breathlessness and underwent a chest enhanced computed tomography (CT) on April 17, 2023. Imaging revealed a 35mm×24mm×27mm lobulated soft tissue mass with heterogeneous enhancement in the dorsal segment of the right lower lobe of the lung along with stretched adjacent interlobar pleura. Further evaluation of radionuclide bone imaging revealed no abnormalities. Tumor marker detection revealed elevated levels of Cyfra 21-1 (7.1ng/mL), SCC (35.0ng/mL), and PROGRP (69.90 pg/mL), while CEA (2.71ng/mL) level was within normal limits (**Figure 1**). Subsequent needle biopsy of the right lung mass confirmed the diagnosis of invasive lung adenocarcinoma with low programmed cell death ligand 1 (PD-L1) expression (tumor proportion score (TPS) < 1%) and harboring mutations in *ERBB2* exon20 p.Y772_A775 dup with frequency of 79.98% and *TP53* IVS9 c.993 + 2T with frequency of 69.52%. The patient first presented to our hospital and underwent positron emission tomography (PET)-CT on May 22, which showed the primary lung lesion (3.5 cm, SUVmax 11.0), bilateral hilar and mediastinal lymph node metastases (2R, 4R, 7, 8), and a 1.4 cm metastatic lesion in the left hepatic lobe (SUVmax 4.7) (**Figure 1**). The patient was staged as cT2aN3M1b and IVA. Four cycles of immunochemistry treatments with sintilimab in combination with pemetrexed and cisplatin were initiated on June 7, with a 10% reduction in chemotherapy dose due to the patient’s poor pulmonary function and Cancer and Aging Research Group (CARG) score of 4. Despite an initial response of stable disease (SD) according to the Response Evaluation Criteria in Solid Tumors (RECIST, version 1.1), disease progression was observed after two subsequent cycles of sintilimab maintenance therapy. On November 9, an enhanced CT scan revealed enlargement of the lung lesion to 6.2 cm×2.7 cm, metastatic lymph nodes, the liver lesion to 5.4 cm×5.2 cm, and a newly discovered right pleural effusion. In addition, head-enhanced magnetic resonance imaging (MRI) revealed the presence of a new brain metastasis. Of note, the patient also developed hypothyroidism, probably related to ICIs therapy, which was treated with hormone supplementation.

Due to the disease progression, the patient was subsequently treated with four cycles of DS-8201 (270 mg, every 3 weeks), which was started on November 17, after exclusion of contraindications. Surprisingly, the lung lesion was reduced by 50% to 3.1 cm×1.7 cm after two cycles, achieving a partial response (PR) and maintaining PR along after four cycles.

On February 23, the patient developed a transient fever of 39.5°C and improved with antibiotics. Upon returning to our hospital on March 6 for the next treatment cycle, the patient complained of severe anorexia, fatigue, and shoulder pain, with an ECOG score of 2, height of 162cm, weight of 42kg, and hyponatremia (Na^+^ 132mmol/L). Blood analysis revealed an elevated level of lymphocytes proportion of 41.5% (March 6) to 50.7% (March 11) and a declined neutrophil-to-lymphocyte ratio (NLR) of 0.89 (March 6) to 0.81 (March 11). Further investigations revealed low serum total cortisol (<0.50μg/dL) and adrenocorticotropic hormone (ACTH) (<3.00 pg/mL). Due to a history of immune-related adverse event (irAE) and a 5-month cessation of ICI therapy, delayed ICI-associated adrenal insufficiency was considered. Prompt treatment with prednisone resulted in symptomatic improvement together with normalization of lymphocytes and NLR, and further MRI evaluation ruled out pituitary lesions or enlargement.

## Discussion

In this case report, we present an elderly patient with *HER2*-positive NSCLC who progressed after first-line immunochemical therapy but demonstrated a remarkable response upon switching to DS-8201 treatment. The dose of DS-8201 administered was 270 mg (equivalent to 6.4 mg/kg). As of the current report, the patient has not experienced any severe adverse events related to DS-8201, such as ILD or neutropenia, and has achieved a PFS of 8 months. Therefore, our plan is to continue treatment with DS-8201 and reduce the dose to 240 mg (approximately 5.4 mg/kg), while closely monitoring the patient for efficacy and safety during follow-up visits.

The ADC agent was designed to consist of a monoclonal antibody, a cytotoxic agent (payload), and a linker to facilitate their conjugation (17). The antibody targets specific tumor cell surface proteins to ensure high tumor specificity, while the Fc region can bind to effector cells with Fcγ receptors, including NK cells, monocyte-macrophages, and neutrophils, thereby activating the antibody-dependent cellular cytotoxicity (ADCC). In addition, the cytotoxic payload can diffuse from the targeted tumor cells to neighboring cells with limited or absent target expression, eliciting an anti-tumor effect known as the “bystander effect”, which is critical to effectively combat heterogeneous tumors.

The breakthrough of DS-8201 can be attributed to its innovative drug design. The payload of DS-8201 functions as a potent topoisomerase I inhibitor with superior membrane permeability compared to trastuzumab emtansine. Extensive *in vitro* and *in vivo* experiments have demonstrated its potent anti-tumor activity through the bystander effect (18). Notably, *in vivo* testing has shown that the bystander effect of DS-8201 does not affect distant cells from the inoculated *HER-2* target, providing evidence of the drug’s safety profile (18).

In 2019, DS-8201 made a spectacular appearance by achieving durable anti-tumor activity in previously treated *HER2*-positive breast cancer in the DESTINY-Breast 01 trial (14). The subsequent DESTINY-Breast series confirmed that DS-8201 can delay disease progression in patients with *HER2*-positive metastatic breast cancer, even including those with low *HER2* expression (19). During the same period, DS-8201 also demonstrated satisfactory efficacy in the second-/third-line treatment of advanced gastric cancer in the DESTINY-Gastric series trials (13, 20). Along with the researches in the pan-cancer field (21), research on the application of DS-8201 in lung cancer is still in its nascent stages, with limited reports, mostly isolated cases (22–27). A literature search was conducted in May, 2024, and relevant case reports were summarized in **Table 1**. Most of these real-world case reports administered DS-8201 to patients after multiple lines of treatment, and achieved good efficacy with most patients experiencing PFS of more than 6 months. However, adverse events such as myocarditis, myelosuppression, and ILD were reported in some cases.

**Table 1:** Summary of case reports applicating DS-8201 in patients with NSCLC.

Study	Age/Gender	Stage	*HER2*	Previous treatment	Line	PFS	AE
Kato (22) (2021)	62/F	IIIB	20ins & Amp	Chemoradiation, Chemo+ICI, Chemo	5L	>6m	Non
Riudavets (23) (2022)	69/F	IV	20ins	Chemo+ICI	2L	6m	AMC
Riudavets (23) (2022)	57/M	IV	20ins	Chemo, ICI	3L	3m	AMC
Xu (24) (2023)	58/M	IV	20ins	Chemo+Bev, Chemo+ICI, Afa, T-DM1, Pyro, et al.	>5L	9m	Myelo-suppression
Wang (25) (2023)	50/F	IV	20ins	Chemo+Bev, Trastu, Pozi, Pyro+ICI, T-DM1, et al.	>5L	10m	Myelo-suppression
He (26) (2024)	52/M	IV	20ins	Chemo	2L	>21m	Myelo-suppression
Nam (27) (2024)	50/F	IV	20ins	Chemo+ICI, Pyro	4L	>10m	ILD

20ins, HER2 exon 20 insertion; Afa, afatinib; AMC, acute myocarditis; amp, HER2 amplification; AE, adverse events; Bev, bevacizumab; Chemo, chemotherapy; ICI, immune checkpoint inhibitor; ILD, interstitial lung disease; PFS, progression-free survival; Pozi, pozitinib; Pyro, pyrotinib; T-DM1, trastuzumab emtansine; Trastu, trastuzumab.

Pending further clinical trials in large populations, DS-8201 has not yet been recommended for the first-line treatment of *HER2*-positive NSCLC. However, some ongoing studies are exploring its potential in combination with immunotherapy, hormone receptor modulators, DNA damage repair inhibitors, and other agents (28). Preclinical models have demonstrated the ability of DS-8201 to modulate the tumor microenvironment (TME) towards an inflammatory phenotype, resulting in increased infiltration of dendritic cells (DCs) and CT8^+^T cells, as well as enhanced expression of PD-L1 and MHC-I molecules on tumor cells (29). Research in *HER2*-positive gastric cancer cell lines has also indicated that DS-8201 has the capacity to modulate immune-related pathways, particularly the cGAS-STING pathway, enhance the gene expression signature of tumor immunogenicity, promote IFN-1 response, and induce DCs activation, which in turn leads to increased tumor cell cytotoxicity (30). From a safety standpoint, while ICIs kill tumors by regulating the immune response, there is a risk of autoimmune damage occurring because of over-activated immune cells, leading to irAEs. This may occur via mechanisms whereby cytotoxic T lymphocytes erroneously target normal tissues that express homologous antigens in a manner analogous to tumor cells, and whereby the upregulation of inflammatory cytokines mediates tissue injury. It is worth noting that some studies have suggested that the occurrence of irAEs may be associated with better immunotherapy efficacy and positive survival outcomes (31, 32). Therefore, it is reasonable to speculate that any factors that facilitate the function of ICI agents may increase the risk of irAEs.

In the case presented, the patient exhibited indications of thyroid toxicity during treatment with sintilimab, followed by a delayed onset of adrenal insufficiency. Adrenal insufficiency is recognized as a rare endocrine irAE. Previous studies have reported the concurrent occurrence of thyroid and adrenal dysfunction, as well as other multiple endocrine irAEs in patients receiving ICIs (33, 34), which may be attributed to the response of T cells within the highly vascularized endocrine system and the complex interactions among various hormonal axes (35). Additionally, the patient experienced several transient episodes of mild neutropenia during the treatment course, while leukocyte, platelet, and hemoglobin levels remained within normal ranges. Each episode of neutropenia was promptly resolved after administration of granulocyte colony-stimulating factor (G-CSF). The patient had no significant prior history of pulmonary or cardiovascular disease, suggesting a favorable safety profile when evaluating for other irAEs such as myocarditis and ILD. Considering the hypothesis that DS-8201 may enhance the efficacy of ICIs through a synergistic effect, we propose that the provocation of delayed irAE in this patient after immunotherapy withdrawal may be potentially related to the activation of TME by DS-8201. This prompts the synergistic potential of combining immunotherapy with DS-8201 to mutually enhance treatment efficacy. However, the lack of evaluation of the status of inflammatory cytokines (such as interleukins and TNF-α), anti-drug antibodies against DS-8201, or specific flow cytometry analysis of lymphocyte subtypes during the March 6 visit, when the patient presented symptoms of adrenal insufficiency, makes it challenging to discriminate whether the observed elevation in lymphocytes was attributed to the recent infection on February 23 or related to an irAE induced by DS-8201. It is essential to monitor these assessments in subsequent patients to determine whether the onset of immune reactions following treatment with DS-8201 is due to acute events, such as infections, or represents a delayed irAE. Furthermore, while our case presents intriguing possibilities, it is critical to acknowledge that a phase Ib trial evaluating the efficacy of DS-8201 in combination with nivolumab versus monotherapy (NCT03523572) presented at ESMO Breast Cancer 2022 did not show a significant improvement, indicating ongoing controversy regarding the benefit of combination regimens.

Moving forward, a deeper understanding of the mechanisms underlying the interactions of DS-8201 and its clinical implications are essential to guide the management of *HER2*-positive cancers. Further mechanistic and clinical research is warranted to elucidate the full potential of DS-8201 in the treatment landscape of *HER2*-positive malignancies.

## Data Availability

The raw data supporting the conclusions of this article will be made available by the authors, without undue reservation.
